# Identification, classification, and characterization of AP2/ERF superfamily genes in Masson pine (*Pinus massoniana* Lamb.)

**DOI:** 10.1038/s41598-021-84855-w

**Published:** 2021-03-08

**Authors:** Peihuang Zhu, Yu Chen, Jinfeng Zhang, Fan Wu, Xiaofeng Wang, Ting Pan, Qiang Wei, Yanping Hao, Xuelian Chen, Chunwu Jiang, Kongshu Ji

**Affiliations:** 1 Key Laboratory of Forestry Genetics & Biotechnology of Ministry of Education, Nanjing, 210037 China; 2 Co-Innovation Center for Sustainable Forestry in Southern China, Nanjing Forestry University, Nanjing, 210037 China; 3 Anhui Academy of Forestry, Hefei, 230031 China

**Keywords:** Molecular biology, Plant sciences

## Abstract

Transcription factors (TFs) play crucial regulatory roles in controlling the expression of the target genes in plants. APETALA2/Ethylene-responsive factors (AP2/ERF) are part of a large superfamily of plant-specific TFs whose members are involved in the control of plant metabolism, development and responses to various biotic and abiotic stresses. However, the AP2/ERF superfamily has not been identified systematically in Masson pine (*Pinus massoniana*), which is one of the most important conifer in southern China. Therefore, we performed systematic identification of the AP2/ERF superfamily using transcriptome sequencing data from Masson pine. In the current study, we obtained 88 members of the AP2/ERF superfamily. All PmAP2/ERF members could be classified into 3 main families, AP2 (7 members), RAV (7 members), ERF (73 members) families, and a soloist protein. Subcellular localization assays suggested that two members of PmAP2/ERF were nuclear proteins. Based on pine wood nematode (PWN) inoculated transcriptome and qPCR analysis, we found that many members of PmAP2/ERF could respond to PWN inoculation and PWN related treatment conditions in vitro. In general, members of the AP2/ERF superfamily play an important role in the response of Masson pine responds to PWN. Furthermore, the roles of the AP2/ERF superfamily in other physiological activities of Masson pine remain to be further studied.

## Introduction

Transcription factors (TFs) play crucial regulatory roles in controlling the expression of the target genes in plants^1^. The AP2/ERF family is a large plant-specific TF superfamily, and has received increasingly more attention by researchers^2^. AP2/ERF TFs contain one or two AP2 domains consisting of around 60 aas^3^. Two classification methods for the AP2/ERF superfamily, which were first proposed by Sakuma et al.^4^ and Nakano et al.^3^ respectively, have been widely used for dozens of plants.

AP2/ERF TFs have been shown to play crucial roles in plant growth and development^5,6^, biotic and abiotic stress responses^7^, and primary and secondary metabolite regulation^8,9^. Li et al. reported that *LeERF1* positively mediated tomato fruit ripening and softening, and an-tisense *LeERF1* transgenic fruits had longer shelf life than the wild-type tomato^10^. Liao et al. revealed that overexpression of a *AP2/ERF* TF(*MsDREB6.2*) may influence stomatal, root then enhanced drought tolerance in apple plants^11^. Li et al. reported that CitERF71 could bind to ACCCGCC and GGCGGG motifs in the promoter of E-geraniol synthetase (*CitTPS16*) and activate the expression of *CitTPS16* in sweet orange fruit^12^. Charfeddine et al. revealed that overexpression of *StERF94* improved the resistance to *Fusarium solani* infection by enhancing expression of pathogenesis related proteins in potato^13^. Plant genomic and transcriptomic sequence data have helped in the identification of AP2/ERF superfamily members in over 50 plant species, including *Arabidopsis thaliana*^3^, rice^3^, *Eucalyptus grandis*^14^, *Populus trichocarpa*^15^, *Triticum aestivum*^16^, and *Hevea brasiliensis*^17^*.*

Masson pine (*Pinus massoniana*) is an economically and ecologically important evergreen conifer in southern China and is one of the most important forest tree species^18,19^. It is widely used in the production of solid wood and resin^20^. Masson pine often suffers abiotic and biotic stress during its life cycle^21^. Pine wood nematode (*Bursaphelenchus xylophilus*) is the main causative pathogen, and PWN disease is a damaging disease that jeopardozes pine, included Masson pine^22^. Liu et al. reported that some members of the *AP2/ERF* superfamily had higher expression in PWN resistant plants than in susceptible Masson pine plants, suggesting that the *AP2/ERF* superfamily may be related to PWN resistance^23^. Moreover, many previous studies revealed that diverse members of group IX and X of ERF TFs from the AP2/ERF superfamily coordinate stress signaling with wound repair and defense response^2,24^.

Because the size of the Masson pine genome is very large, possibly more than 20 Gb, the identification of *AP2/ERF* superfamily genes in Masson pine has not been carried out, but it is very necessary. So we identified the AP2/ERF superfamily members based on four Masson pine transcriptomic datasets. Then the phylogenetic grouping and protein motif structural analyses were investigated to classify the PmAP2/ERF superfamily via the *A. thaliana* AP2/ERF superfamily classification as a reference. Furthermore, several genes of *AP2*/*ERF* were subjected to qPCR analysis, to test there expression patterns in Masson pine needles under treatments in vitro associated with pine wood nematode (PWN). In this study, we identified and classified the *AP2/ERF* superfamily genes in Masson pine, lays a foundation for future study of *AP2/ERF* and facilitates future research on the responses to environmental stresses in Masson pine.

## Results

### Identification and phylogenetic grouping of AP2/ERF family proteins in Masson pine

We identified a total of 88 putative full-length AP2/ERF family genes (*PmAP2/ERF1- PmAP2/ERF88*, Pm is the abbreviation for *Pinus massoniana*) from Masson pine (Table S1). Among the proteins encoded by these genes, 7 containing two AP2 domains each were annotated as AP2 family TFs, while 7 containing a single AP2 domain along with a B3 domain were grouped into the RAV family. Seventy-four proteins containing a single AP2 domain were annotated as ERF family members (73 in total), plus one soloist protein.

**Figure 1:**
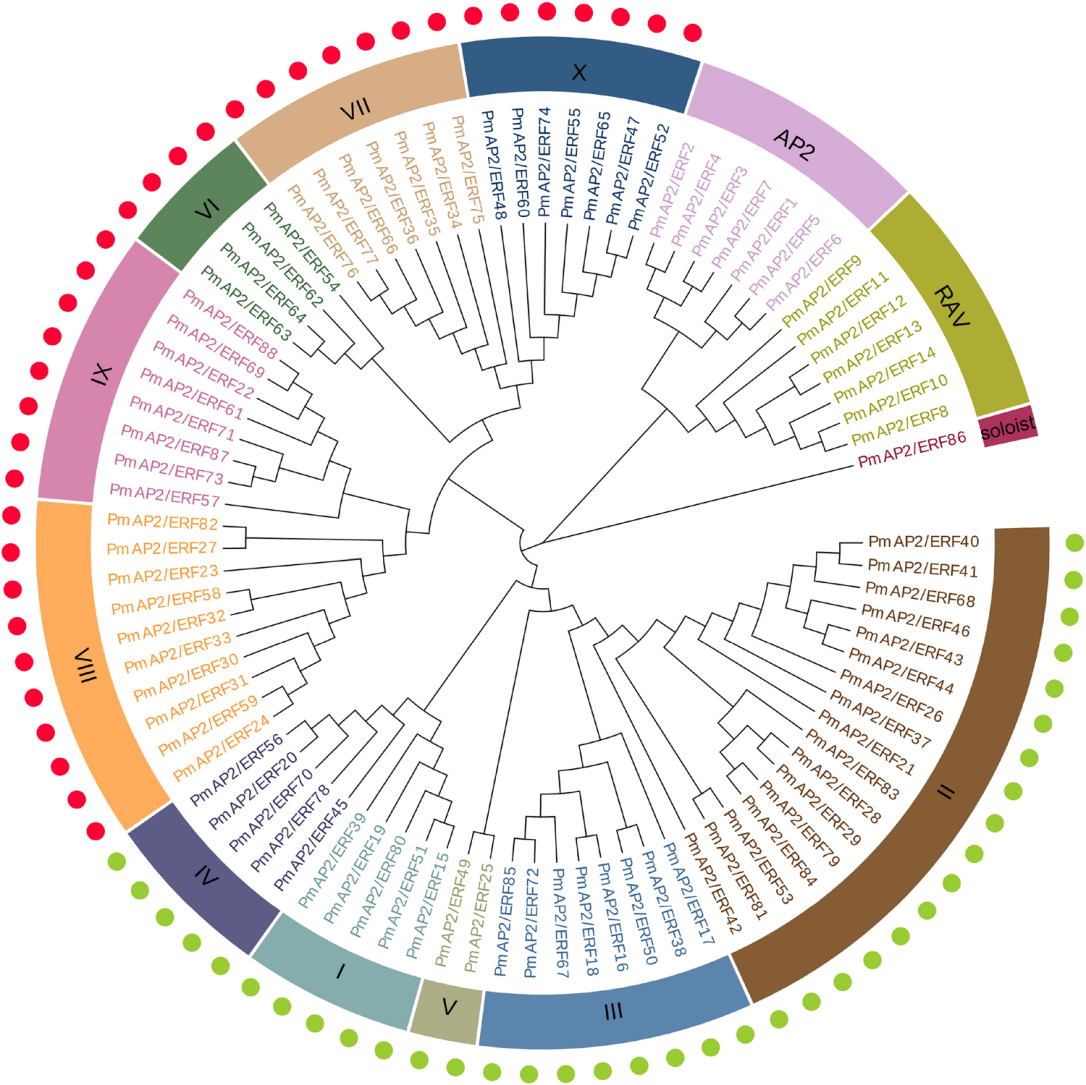
Phylogenetic grouping of AP2/ERF superfamily members in *P. massoniana*. AP2 family, RAV family and ten clades (I-X) of ERF family were shown in a different coloured circle strip. DREB and ERF subfamilies were shown using green and red dots respectively. phylogenetic tree was constructed using MEGA X with ML method.

The 88 putative *PmAP2/ERF* genes encode proteins that range from 138 to 822 aas in length and whose molecular weight ranges from 15.24 to 101.24 kDa (Table S2). The aa length of ERF family members is between 138 and 532, while that of all the AP2 and RAV subfamily members is longer than 418 (Table S2). The pI values of the predicted AP2/ERF TFs ranged from 4.63 to 11.61, with 38 (43.18%) proteins having pI values > 7 (Table S2). To classify the evolutionary relationships of the PmAP2/ERF family members in Masson pine, the full-length aa sequences of the putative proteins were aligned, and a phylogenetic tree was constructed using AP2 domain aa sequences cut from aligned full-length aa sequences (Fig. 1). Based on the phylogenetic tree of the PmAP2/ERF family members, seven AP2 family members were named PmAP2/ERF1-PmAP2/ERF7, seven RAV family members were named PmAP2/ERF8-PmAP2/ERF14, and 73 ERF members and one soloist protein were named PmAP2/ERF15-PmAP2/ERF88.

According to the classification of the ERF family in *A. thaliana*^3^, 73 ERF members were classified into ten groups, namely, groups I to X (Fig. 1). Groups I to X contain 5, 17, 8, 5, 2, 4, 7, 10, 8 and 7 members, respectively. With a few exceptions, most of the clades contain members from both species, indicating a common origin (Figure S1). According to previous works of Sakuma et al.^4^, the ERF family comprises two major subfamilies: ERFs and DREBs. Both groups could be clearly demarcated in the tree, with groups I to V corresponding to the DREB subfamily (37 members, marked by green dots) and groups VI to X corresponding to the ERF subfamily (36 members, marked by red dots) of the ERF family (Fig. 1).

### Conserved motifs of PmAP2/ERF superfamily TFs

**Figure 2:**
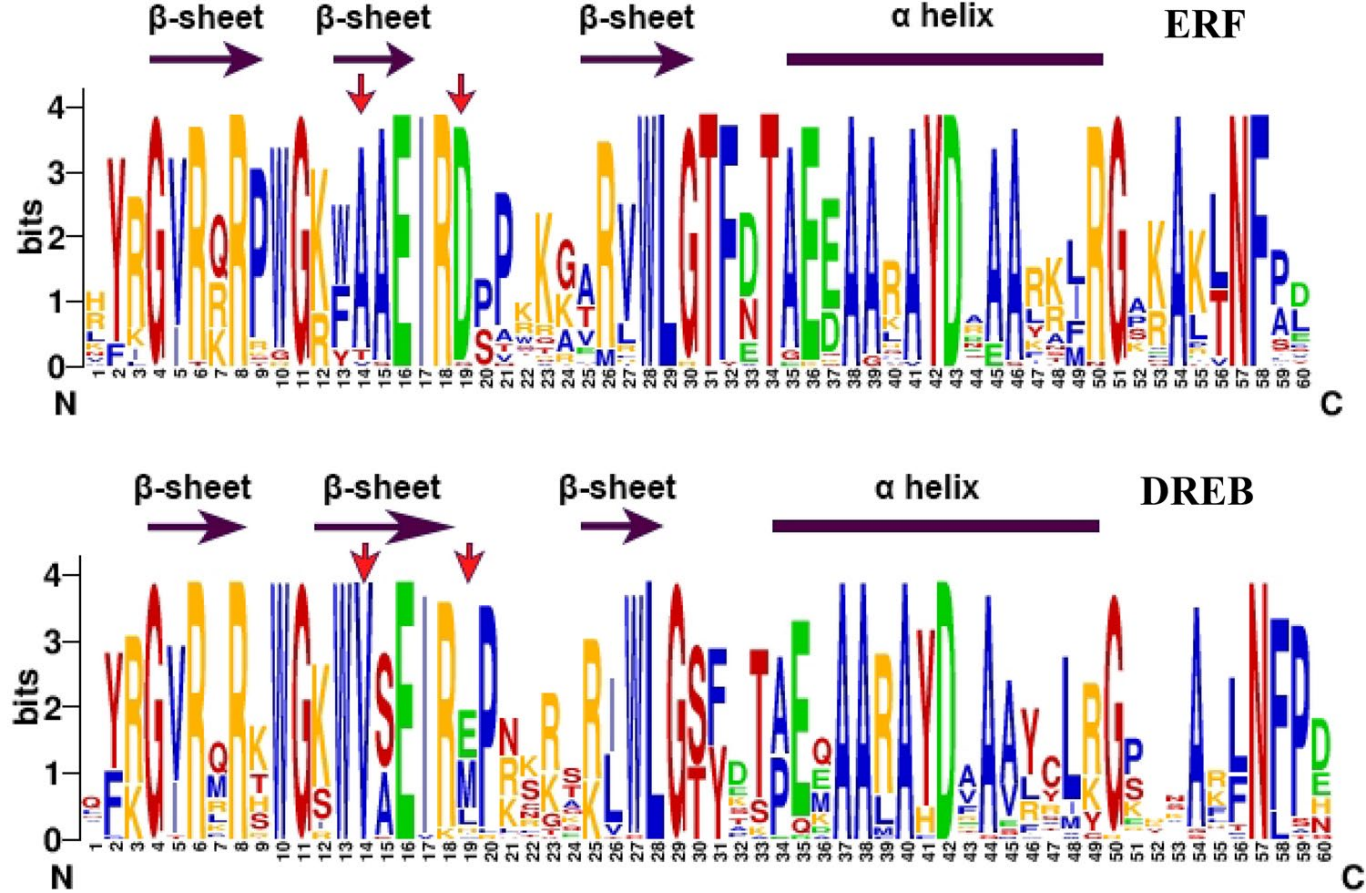
Sequence logo of AP2/ERF domains extracted from ERF and DREB protein sequences information. The β-sheets and α-helix were marked using purple arrows and boxes respectively. Specific conservated aa residues in the second β-sheet from ERF and DREB protein sequences were marked by red arrows.

To analyze the conservation of the predicted PmAP2/ERF superfamily TFs, the deduced aa sequences of the AP2 domains were aligned with those of the corresponding AP2/ERF proteins in *A. thaliana*, which have been identified previously (Figure S2). With the exceptions of PmAP2/ERF50 (group III, DREB subfamily) and PmAP2/ERF73 (group IX, ERF subfamily), which contain OLR and WLR aa residues at the same site, respectively, all of the predicted aa sequences of the AP2 domain of the ERF family proteins have a WLG motif. Among the members of the ten ERF family groups, the members of groups I, V and VII exhibited a higher level of conservation compared with those of the other groups (Figure S2). Because the ERFs and DREBs had slightly different aa sequences in their AP2 domains^25^, we built two sequence logos for AP2 domains respectively from 36 ERFs and 37 DREBs (Fig. 2). Each sequence logo contained three β-sheets and an α-helix (Fig. 2). The A14 and D19 aa residues in the second β-sheet exhibited conservation of most of the ERF sequences, as marked by red arrows (Fig. 2)^2^. Similarly, among the DREBs, V14 is largely conserved, while position 19 is conserved across groups: L19 in group I, E19 in groups III and IV, and M19 in group II. Moreover, glutamic acid and methionine residues generally have a high frequency at position 19. The AP2 domain of 73 ERF family members had 11 absolute conserved aa sites: G4, R8, G11, E16, R18, W28, L29, A38, A41, D43 and G51. Moreover, there were 9 conserved sites: R3 (70/73), R6 (72/73), W10 (70/73), I17 (72/73), G30 (72/73), E36 (69/73), A39 (71/73), A55 (70/73) and N58 (72/73). These conserved aa residues are usually associated with DNA binding (Figure S3). PmAP2/ERF1-PmAP2/ERF7 contain two AP2 domains; therefore, they are classified into the AP2 family, and PmAP2/ERF8-PmAP2/ERF14 contain an AP2 domain and a B3 domain; thus, they were assigned to the RAV family. The AP2 domains of Masson pine RAV and AP2 family members share many conserved aa residues with those of *A. thaliana*. The WLG motif in the RAV subfamily in Masson pine is highly conserved, while in the AP2 subfamily, the motif is instead presented as YLG (Figure S2).

**Figure 3:**
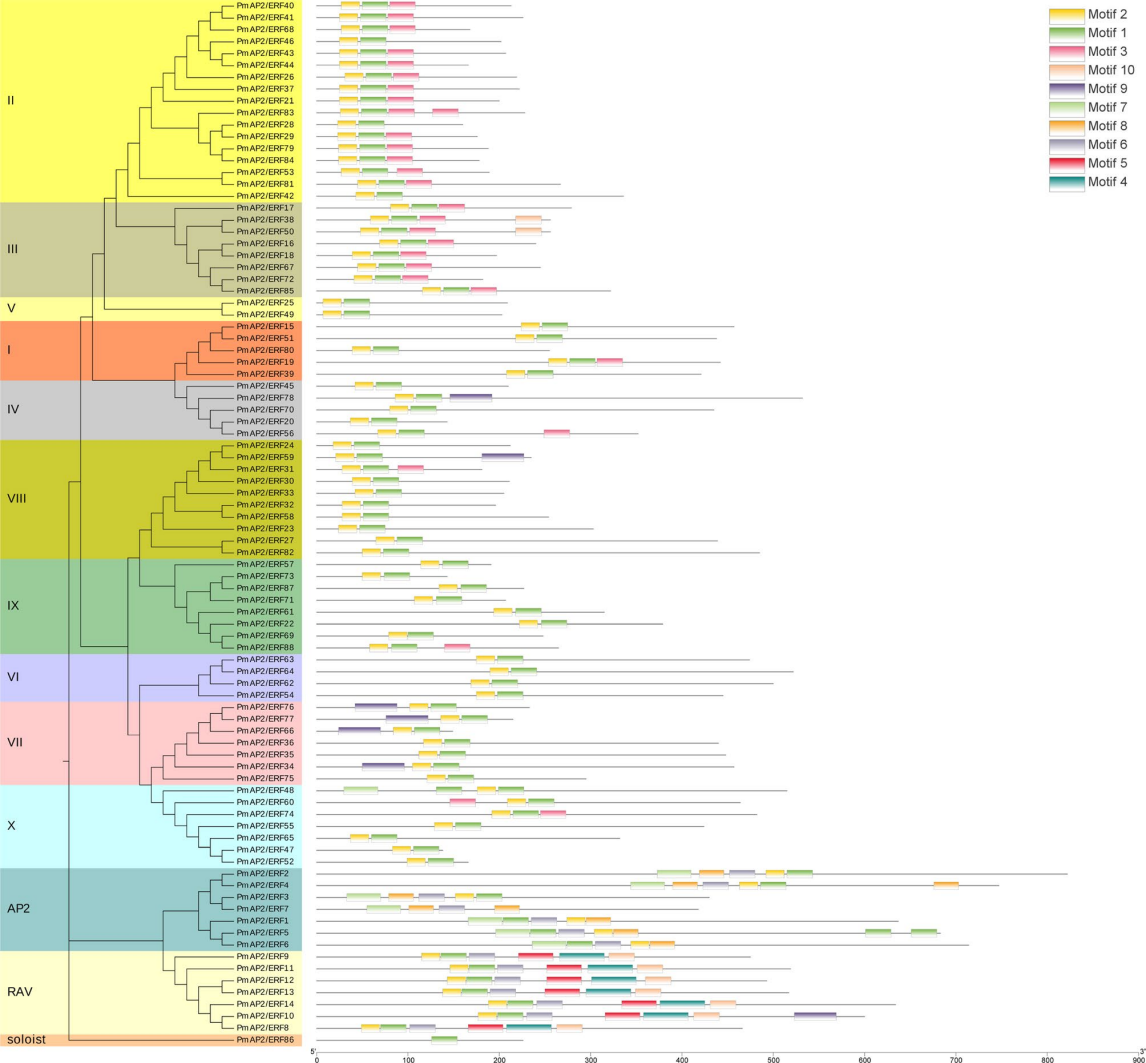
Phylogenetic grouping and motif distribution of AP2/ERF proteins in *P. massoniana.* Ten conserved motifs identified by MEME are indicated by different coloured boxes, and their relative positions are displayed. Details of each motif were shown in supplementary Figure S4.

The reliability of the phylogenetic grouping of PmAP2/ERF family proteins was further supported by motif analysis using the MEME motif analysis tool (Fig. 3). A total of 10 motifs were identified from PmAP2/ERF family TFs, and for each motif, a separate sequence logo was generated (Figure S4). Motifs 1 and 2 correspond to the AP2 domain and were conserved in ERF and RAV families, while two adjacent AP2 domains contained additional motifs (7 and 8). Motifs 4 and 5 correspond to the B3 domain in the RAV family. Overall, the motif organization of most of the members belonging to a particular clade is similar, such as motif 3 being exclusively present in groups II and III, and motifs 18, 15, 12 and 10 being exclusive to groups II, III, IV, and VII, respectively. These results indicated the coevolution of the AP2 domain with the rest of the protein sequence.

### Subcellular localization of PmAP2/ERF superfamily proteins

**Figure 4:**
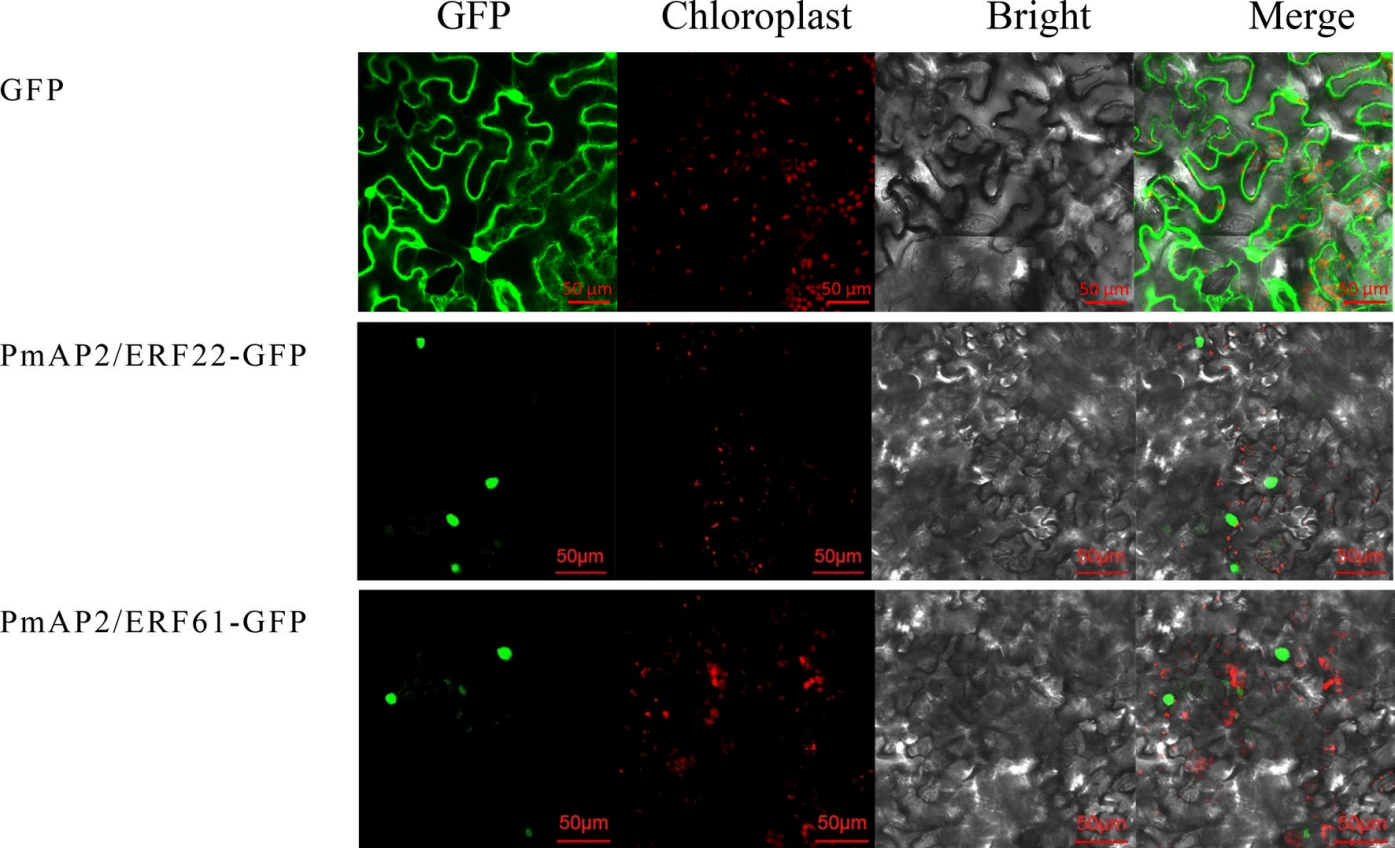
Results of subcellular localization assays of PmAP2/ERF22 and PmAP2/ERF61 in *N. benthamiana* leaves.

After the conserved domains of PmAP2/ERF family TFs were determined, PSORT and CELLO were used for subcellular localization prediction, and 87 PmAP2/ERFs (excluding PmAP2/ERF33) had the same predicted localization according to both methods. Of these, 77 PmAP2/ERFs were located in the nucleus, and 8 were located at more than one site. PmAP2/ERF79 and PmAP2/ERF20 were located in the chloroplasts and mitochondria, respectively (Table S3). In addition, two genes were selected for an instantaneous expression experiment to further explore the subcellular localization characteristics of the PmAP2/ERF family members. Fluorescent signals of PmAP2/ERF22 and PmAP2/ERF61 were observed in the nucleus (Fig. 4).

### Gene expression analysis via transcriptome data of PmAP2/ERFs during pine wood nematode inoculation treatments

To evaluate the potential function of PmAP2/ERF genes in response to pine wood nematode inoculation, we investigated the gene expression patterns based on the transcriptome data.

**Figure 5:**
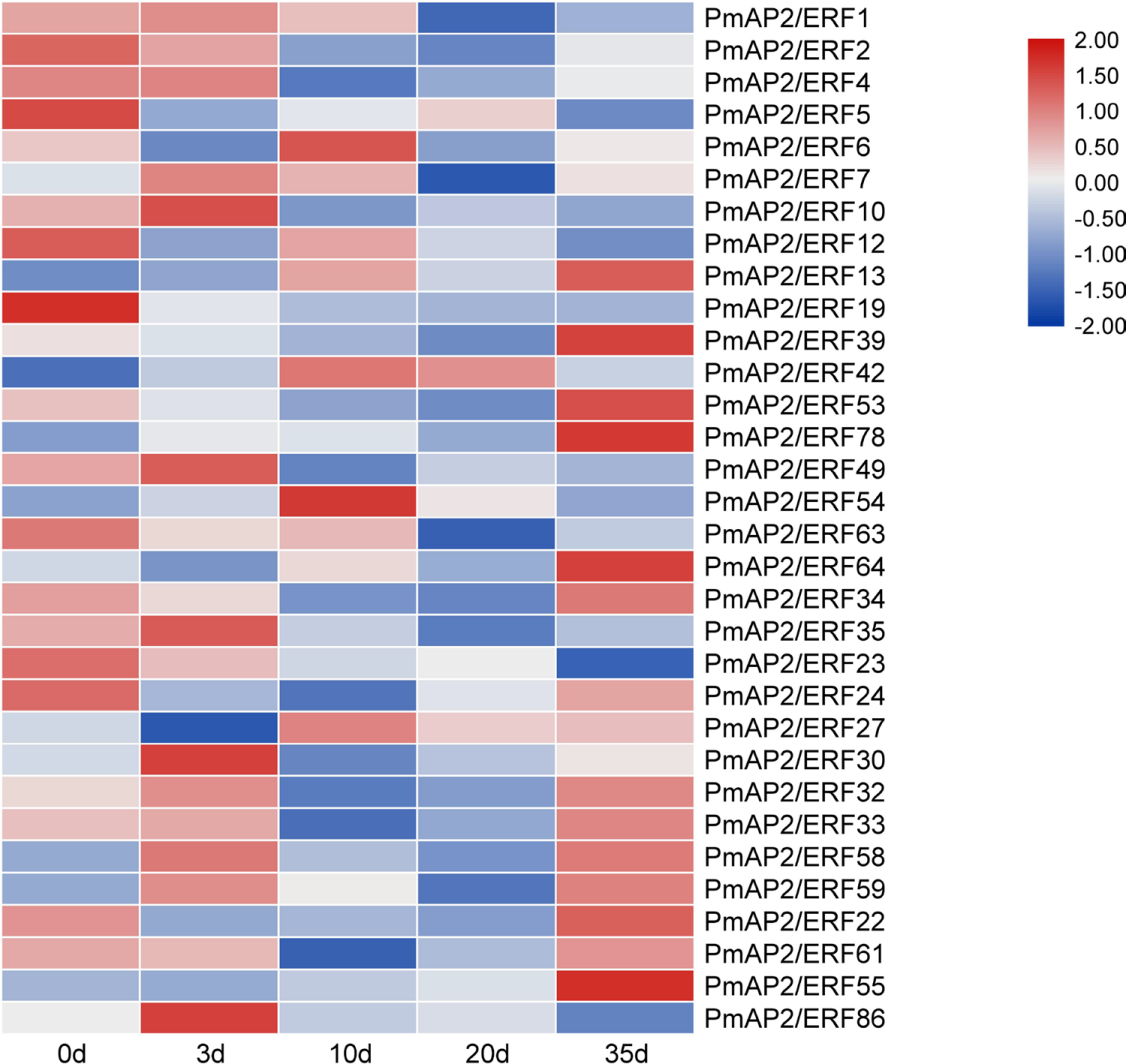
Transcriptional profile of *AP2/ERF* genes during pine wood nematode inoculation treatments in *P. massoniana* at five different stages: 0 day, 3 day, 10 day, 20 day and 35 day. Heat maps were generated using log2(FPKM + 1) values, then performed row scale. Dark blue indicates a low expression level, light color indicates a medium level, and red indicates a high level.

The results show that 32 PmAP2/ERFs belonging to the AP2, RAV, and ERF families and soloist protein were transcribed during pine wood nematode inoculation (Fig. 5). The expression of the majority of AP2 and RAV family members decreased after pine wood nematode inoculation, except for PmAP2/ERF13. For the ERF family, the group VIII exhibited the greatest number of members (8 members). No members of the group III were detected. The expression levels of the majority of ERF family members gradually decreased but then increased, and the abundance of most PmAP2/ERFs was low at 20 d. The majority of the groups VIII, IX and X had high expression levels at 35 d, especially PmAP2/ERF55 (group X). The expression level of the soloist gene peaked at 3 d and then continued to decline.

### qPCR-based analysis of *PmAP2/ERF* genes under different treatments

To understand how *PmAP2/ERF* genes participate in the response to different abiotic conditions, we analyzed the expression of *PmAP2/ERF* genes from groups IX and X in Masson pine under injury treatment and in response to ETH, H_2_O_2_, MeJA, and SA at 0 h, 3 h, 6 h, 12 h, and 24 h using qPCR. According to the literature, *PmAP2/ERF* genes from groups IX and X are involved in coordinating stress signaling with wound repair and defense response^2,24^.

**Figure 6:**
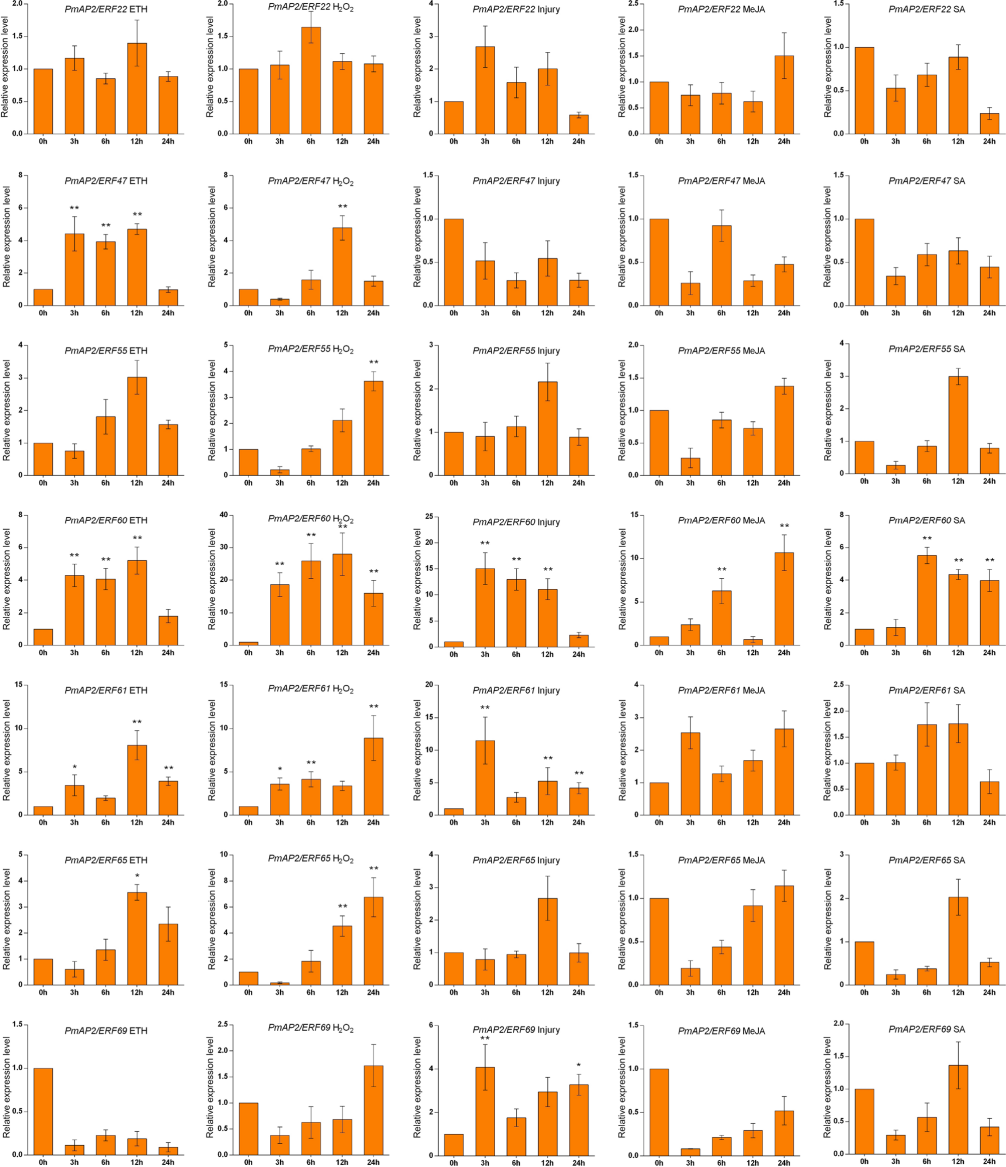
Expression of *AP2*/*ERF* genes in response to different treatments related to pine wood nematode infection in vitro. The asterisks indicate stress the treatment groups that significantly differed with respect to transcript abundance compared with that of the control group (*p < 0.05, **p < 0.01).

The expression of four *PmAP2/ERF* genes in the needles was induced by ETH treatment. The expression of *PmAP2/ERF47* and *PmAP2/ERF60* was upregulated significantly at 3 h, 6 h and 12 h (3.93- to 4.71-fold and 4.06- to 5.20-fold change, respectively), and the expression of *PmAP2/ERF61* was upregulated significantly at 3 h, 12 h and 24 h, especially at 12 h (8.08-fold change). The expression of *PmAP2/ERF65* was upregulated significantly at 12 h (3.56-fold change) (Fig. 6).

The expression of five *PmAP2/ERF* genes was induced in the needles by H_2_O_2_ treatment. The expression of *PmAP2/ERF47* and PmAP2/ERF55 was upregulated significantly at 12 h (4.78-fold change) and 24 h (3.63-fold change), respectively. The expression of *PmAP2/ERF60* was dramatically upregulated at all time points, especially at 12 h (27.99-fold change). Moreover, the expression of *PmAP2/ERF61* was upregulated significantly at 3 h, 6 h and 24 h, especially at 24 h (8.90-fold change), and the expression of *PmAP2/ERF65* was upregulated significantly at 3 h, 6 h and 24 h, especially at 24 h (8.90-fold change) (Fig. 6).

The expression of three *PmAP2/ERF* genes in the needles was induced in response to injury. The expression of *PmAP2/ERF60* was upregulated significantly at 3 h, 6 h and 12 h, especially at 3 h (15.02-fold change). Moreover, the expression of *PmAP2/ERF61* was upregulated significantly at 3 h, 12 h and 24 h, especially at 3 h (11.48-fold change), and the expression of *PmAP2/ERF69* was upregulated significantly at 3 h (4.08-fold change) (Fig. 6).

The expression of *PmAP2/ERF60* was upregulated significantly in the needles at 6 h and 24 h, especially at 24 h (10.66-fold change), in response to MeJA. However, the expression of five *PmAP2/ERF* genes (*PmAP2/ERF22*, *PmAP2/ERF47*, *PmAP2/ERF55*, *PmAP2/ERF65*, *PmAP2/ERF69*) was downregulated after MeJA treatment (Fig. 6).

The expression patterns of the *PmAP2/ERF* genes in response to SA were similar to those in response to MeJA. The expression of *PmAP2/ERF60* was upregulated significantly in the needles at 6 h, 12 h and 24 h, especially at 6 h (5.52-fold change). Moreover, the expression of five *PmAP2/ERF* genes (*PmAP2/ERF22*, *PmAP2/ERF47*, *PmAP2/ERF55*, *PmAP2/ERF65*, *PmAP2/ERF69*) was downregulated after SA treatment (Fig. 6).

## Discussion

Due to their important roles in development, metabolism, and response to various stresses, *AP2/ERF* genes have been identified in many plant species^2,24^. Different gene families play different roles in plants. Members of the AP2 superfamily are associated with plant development, while ERF and DREB family genes are prominent candidates for plant abiotic stress tolerance and biotic stress resistance^2^. Moreover, genes within the same gene family, annotated to specific groups, have also been reported to have different functions^24^. Therefore, it is crucial to classify *AP2*/*ERF* genes in detail, which is important for gaining insight into the functions of *AP2*/*ERF* genes.

The AP2/ERF superfamily in *Arabidopsis* comprises 3 families (AP2, RAV and ERF) and a soloist protein, classified based on sequence similarity, number of AP2 domains, and the characteristic motifs. AP2/ERFs in *Arabidopsis* are classified via two different systems^3,4^, and both classification systems have been used to classify the AP2/ERF family proteins in other plant species, including celery and buckwheat^26,27^. Advancements in sequencing technology and the availability of large datasets have aided the identification of *AP2*/*ERF* family genes in several plant species by the use of genomic, transcriptomic or EST data^28,29^. Current data on the molecular and genetic properties of Pinaceae species are lacking due to the large genome sizes of Pinaceae species, the sizes of which are nearly 20 Gb^30^, and access to the genomic information is limited. Only a few genomic databases have been established for *P. taeda*^31^, *P. abies*^32^, and *P. glauca*^30^ in recent years. The lack of reference genomic data has limited Masson pine research. Transcriptome sequencing is a feasible and economical technique for generating relatively comprehensive sequencing data within a short time, and this technique has become popular in plant research^19,28^. Previous studies have demonstrated that the *AP2*/*ERF* gene family is one of the most abundant transcriptional factor families in Masson pine^19,33^.

AP2/ERF TFs are thought to be involved in the response to drought stress, the response to pine wood nematode disease and the biosynthesis of resin in Masson pine, the most abundant resin-producing species worldwide, but Masson pine is also threatened by pine wood nematode disease^34,35^. For the past few years, Masson pine clones that are highly resistant to pine wilt disease have been screened in southern China^36^. Moreover, Masson pine is a good resource for identifying genes associated with resin biosynthesis and pine wood nematode disease resistance^22,33^. However, few *AP2*/*ERF* genes have been reported in Masson pine until now, and these genes have rarely been reported across Pinaceae species. To our knowledge, the transcriptome-based identification and classification of the AP2/ERF family presented here is the first AP2/ERF family analysis in any Pinaceae species. Thus, it lays a foundation for further functional analysis of *AP2*/*ERF* genes in Masson pine and serves as a reference for the classification of the *AP2*/*ERF* gene family in other Pinaceae species. Notably, the number of identified AP2/ERF members in Masson pine may increase in the future, as the present number of predicted genes is based on transcriptome sequencing data. For example, in this study, we did not find AP2 family genes with a single AP2 domain in Masson pine, while five AP2 family genes have been shown to contain a single AP2 domain in *A. thaliana*^3^.

Phylogenetic grouping revealed the suitability of using the classic AtAP2/ERF-based classification method with Masson pine-derived sequencing data. The AP2 domain of AP2/ERF genes is reportedly highly conserved among different plant species, although the sequence similarity outside the AP2 domain is very low^37^. Like those in other plant species such as pear^38^, *Ammopiptanthus nanus*^39^ and moss^29^, our results showed that the aa composition of the AP2 domain was very conserved between *Arabidopsis* and Masson pine, and the G4, L29, and A38 aas were completely conserved among all ERF members from Masson pine and *A. thaliana*.

Other aa variations can further refine the classification of genes; for example, the HLG and WLG motifs in the third β-sheet of the AP2 domain can distinguish the soloist genes from ERF genes, and the AIK and EIR motifs in the second β-sheet of the AP2 domain have the same effect. Interestingly, we found that position 19 houses an infrequent H19 in both group V members in Masson pine and houses the same H19 in four and six group V proteins from ERFs in *A. thaliana* (AT5G25390, AT5G25190, AT5G11190) and three other Pinaceae species (Pta011080 in *P. taeda*; Psi008723 in *Picea sitchensis*; and MA10430231g0010, MA54341g0010, MA94228g0010, and MA3073g0010 in *P. abies*). This suggests that conserved aa sites in some specific groups may play a key role in the function of the genes in this group. In the present study, based on the H19 site and DREB-specific V14 of group V genes in Masson pine and the fact that their positions are completely within the DREB branch of the phylogenetic tree, we believe the group V genes in Masson pine are a type of DREB rather than an ERF. Group V has been confirmed to be a type of ERF family^3^. This divergence may be due to the different degrees of evolution between Masson pine and *A. thaliana*, while the former is an ancient gymnospermous species. Our results expand upon the idea that AP2/ERF genes in moss species are different from those in angiosperms.

Gene expression patterns are considered to be directly associated with gene function^35^, and in this study, based on transcriptome sequencing data, the transcript abundance of 32 *PmAP2*/*ERF* genes was obtained in the pine wood nematode disease resistance process. These transcripts included members from the AP2 and RAV families as well as nearly all groups of the ERF family. Moreover, the expression of seven ERF family genes from groups IX and X differed in response to ETH, MeJA, H_2_O_2_, SA and injury treatments. The gene expression patterns were different within the same family, suggesting a functional diversity of AP2/ERF genes in response to different treatments. Moreover, while the treatment duration progressed, these significantly differentially expressed genes may be closely related to the signal transduction process in response to the treatment conditions.

In brief, the identification, classification, and expression profiling of the PmAP2/ERF family genes provide insight into the biological functions of the individual AP2/ERF TFs in Masson pine.

## Conclusions

The identification and classification of the AP2/ERF transcription factor superfamily is indispensable in the functional study of *AP2/ERF* genes in Pinaceae plants. However, there are few reports in this field. To supplement the research in this area, we identified and classified the AP2/ERF family of Masson pine for the first time. The results showed that eighty-eight AP2/ERF predicted proteins were identified from four Masson pine transcriptomes. Based on pine wood nematode inoculated transcriptome and qPCR analyses, we found that many members of PmAP2/ERF could respond to PWN inoculation and PWN-related treatment conditions in vitro. These *PmAP2/ERF* genes are promising candidate genes for further functional analysis and demonstrate great potential for breeding efforts related to pine wood nematode disease resistance in Masson pine.

## Materials and methods

### Identification of the AP2/ERF family in Masson pine

The unigene sequences of Masson pine were derived from the previously determined CO_2_ stress transcriptome^19^, young shoots transcriptome (PRJNA655997), drought stress transcriptome(SRA accession: PRJNA595650) and pine wood nematode inoculated transcriptome(SRA accession: PRJNA660087). The hidden Markov model (HMM) of the Apetala 2 (AP2) (PF00847) was downloaded from the Pfam database (http://pfam.xfam.org/), and the AP2/ERF family TFs in Masson pine were identified using HMMER3 (v3.0) software with a defined threshold of E < 1E^−3^. Pfam and NCBI Conserved Domain Search (CD Search) (https://www.ncbi.nlm.nih.gov/Structure/cdd/wrpsb.cgi) were used to manually confirm the predicted AP2/ERF family TFs after removing redundant sequences with 95% similarity between different databases, and only sequences longer than 130 amino acid residues were retained for further analysis.

### Sequence analysis, subcellular localization and phylogenetic tree construction

Multiple alignments of aa sequences were performed using MEGA 7.0^40^, and the AP2 domain region was selected. A phylogenetic tree comprising families and groups was constructed based on the AP2 domain sequences of PmAP2/ERF proteins from *A. thaliana* and Masson pine using the maximum likelihood (ML) method with 1000 bootstrap replicates via MEGA X^41^.

The protein theoretical molecular weight and isoelectric point (pI) were analyzed using the ProtParam program (https://web.expasy.org/protparam/). The subcellular localization of PmAP2/ERF proteins was predicted using the PSORT (https://psort.hgc.jp/) and CELLO programs (http://cello.life.nctu.edu.tw/). PmAP2/ERF-22 and PmAP2/ERF-61 were selected for transient transformation. The primers used for vector construction are shown in Table S4. The open reading frame (ORF) regions were inserted into a pCAMBIA-1302-GFP expression vector harboring a green fluorescent protein (GFP). Transient expression vectors (35S::PmAP2/ERF-22-GFP and 35S::PmAP2/ERF-61-GFP) were subsequently constructed; these vectors were transformed into *Agrobacterium tumefaciens* strain GV3101. Both strains along with a p19 (an RNA silencing repressor) *Agrobacterium* strain were grown in LB media for 36 h at 28 ℃ and then suspended in infiltration media (10 mM MgCl_2_, 10 mM 2-(N-morpholino)ethanesulfonic acid (MES), 150 μM acetosyringone). For transient transformation, the suspension cells and p19 were mixed together at a 1:1 ratio, after which the mixture was coinfiltrated into leaves of 30- to 40-days-old *Nicotiana benthamiana* plants. The infiltrated tobacco plants were maintained in a growth chamber at 22 °C with 16 h light/8 h dark photoperiod for 24 h. The GFP signals of the tobacco leaves were captured with an LSM710 confocal laser scanning microscope (Zeiss, Jena, Germany).

### Identification of conserved domains in PmAP2/ERF proteins

The deduced aa sequences of the AP2/ERF proteins were analyzed with MEME 5.1.1 (http://meme-suite.org/tools/meme), with the following parameters: optimum width of 6–60 aas for the motif, and a maximum number of motifs of 10. The sequences of the conserved domain were generated using the online WebLogo platform (http://weblogo.berkeley.edu/).

### Transcriptional profile analysis of *PmAP2/ERF* genes

With respect to *PmAP2/ERF* gene expression analysis, RNA sequencing (RNA-seq) data were obtained from a transcriptome in response to pine wood nematode inoculation. The expression level of the *PmAP2/ERF* genes was calculated by determining the number of expected fragments per kilobase of transcript per million mapped reads (FPKM). Heat maps were generated using log2(FPKM + 1) values, and analyses were performed at the row scale.

### Plant material, RNA extraction and qPCR-based analysis

Two-year-old Masson pine seedlings were transplanted into pots containing a soil mixture (peat:perlite:vermiculite, 3:1:1 (v/v)) under conditions of 24 ℃ and a 16-h light/8-h dark photoperiod. Seedlings at similar growth stages were selected for subsequent treatment.

Five treatments were applied in this in vitro study: mechanical injury, 10 mM H_2_O_2_, 100 µM methyl jasmonate (MeJA), 500 μM ethephon (ETH) and 1 mM salicylic acid (SA). The seedlings were exposed to the treatments for 0 h, 3 h, 6 h, 12 h, 24 h and 48 h, and the leaves were taken as samples for RNA extraction. Samples at 0 h were used as controls. Each treatment was repeated three times independently as biological replicates. All the collected samples were immediately frozen in liquid nitrogen and then stored at − 80 ℃ until RNA isolation.

Total RNA was extracted using a DP441 RNAprep Pure Kit (Tiangen Biotech, Beijing, China) in conjunction with a gDNA-removal step according to the manufacturer′s instructions. The RNA concentration and purity were measured with a NanoDrop 2000 instrument (Thermo Fisher Scientific, Waltham, Massachusetts, USA), and the RNA integrity was estimated via 1.2% agarose gel electrophoresis. cDNA (20 μL) was synthesized from 1000 ng of total RNA using a first-strand cDNA synthesis kit (11141, Yeasen Biotech, Shanghai, China) according to the manufacturer′s instructions.

qPCR was performed via a StepOne Plus System (Applied Biosystems, Foster City, USA) using SYBR Green Real-time PCR Master Mix (QPK-201, Toyobo Bio-Technology, Shanghai, China). Alpha-tubulin (TUA) was used as an internal control gene^35^. The gene-specific primers used for qPCR are shown in Table S4. Each PCR mixture (10 μL) consisted of 1 μL of diluted cDNA (20 × dilution), 5 μL of SYBR Green Real-time PCR Master Mix, 0.4 μL of each primer (10 μM), and 3.2 μL of ddH_2_O. The amplification conditions were as follows: 95 ℃ for 60 s for predenaturation, 40 cycles at 95 ℃ for 10 s for denaturation, and 58 ℃ for 30 s for annealing and extension. Melting curves were analyzed from 60 to 95 ℃ to confirm primer specificity and lack of primer dimers. Each reaction was repeated three times. Negative controls were included on each plate and for each sample, with ddH_2_O and total RNA in place of cDNA. The relative expression levels were calculated according to the 2^−ΔΔCt^ method^42^.

## Supplementary Information

Supplementary Information 1.

Supplementary Information 2.
