# Supplementary material for: Identification, classification, and characterization of AP2/ERF superfamily genes in Masson pine (Pinus massoniana Lamb.)
Source: Sci Rep. 2021 Mar 8;11:5441. doi: 10.1038/s41598-021-84855-w (PMC7940494; doi:10.1038/s41598-021-84855-w)
Supplement: Supplementary file 1 — Supplementary Information 1. [file 41598_2021_84855_MOESM1_ESM.doc]

**Identification, Classification, and Characterization of AP2/ERF Superfamily Genes in Masson Pine (*Pinus massoniana* Lamb.)**

Peihuang Zhu^1,2^, Yu Chen^1,2^, Jinfeng Zhang^1,2^, Fan Wu^1,2^, Xiaofeng Wang^1,2^, Ting Pan^3^, Qiang Wei^3^, Yanping Hao^3^, Xuelian Chen^3^, Chunwu Jiang^3^, Kongshu Ji^1,2,*^

1. Key Laboratory of Forestry Genetics & Biotechnology of Ministry of Education, Nanjing 210037, China
2. Co-Innovation Center for Sustainable Forestry in Southern China, Nanjing Forestry University, Nanjing 210037, China
3. Anhui Academy of Foresty, Hefei 230031, China

***** Correspondence: ksji@njfu.edu.cn; +86-025-8542-7308

| 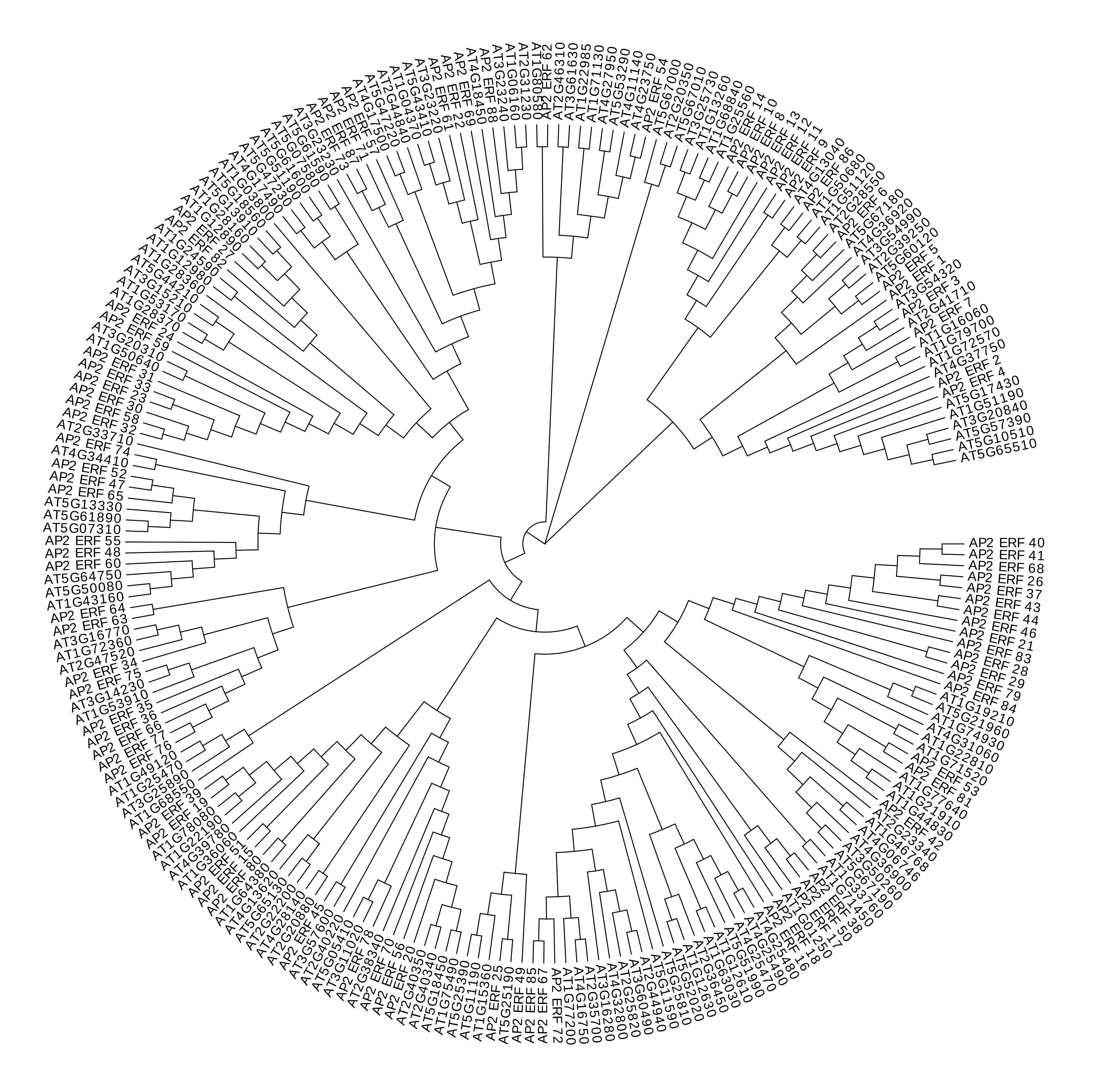 |
| --- |
| **Figure S1.** Phylogenetic analysis of AP2/ERF superfamily members in *P. massoniana*. |

| 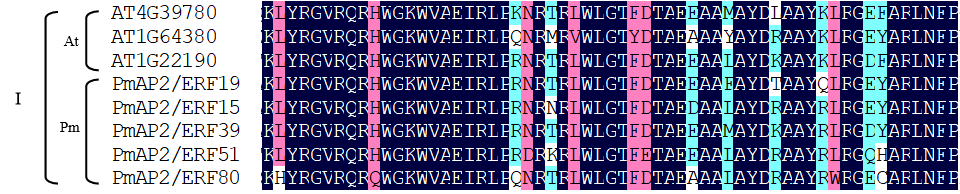 |
| --- |
| 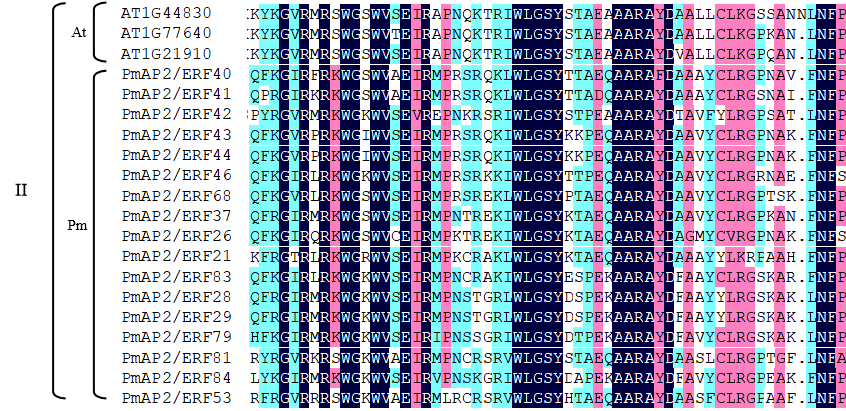 |
| 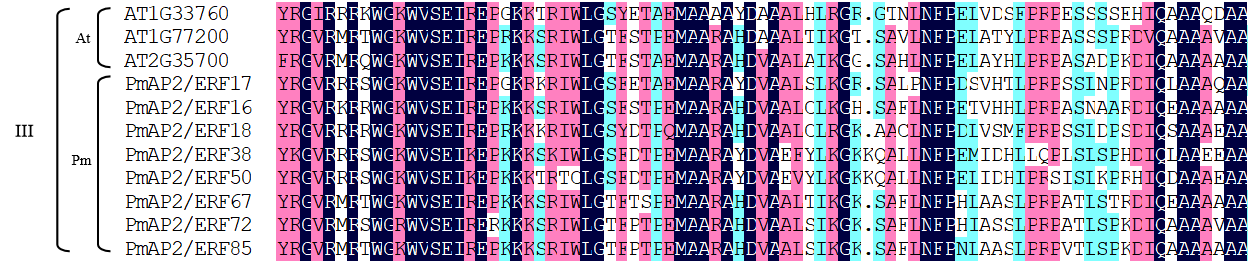 |
| 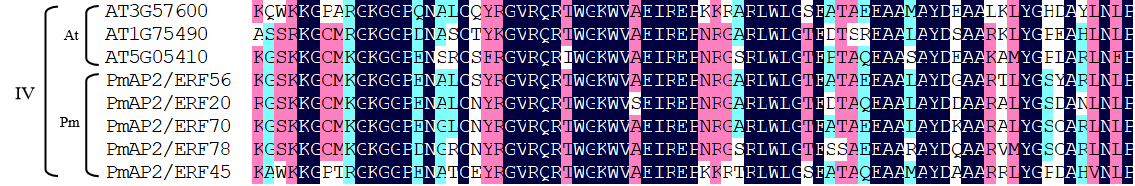 |
| 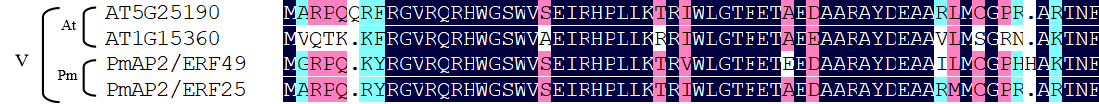 |
| 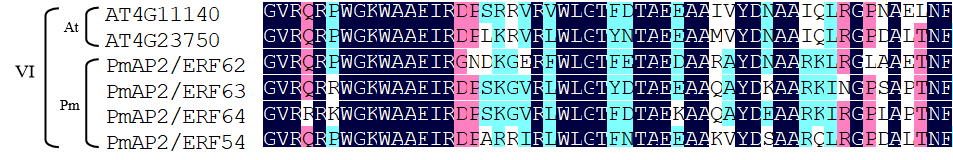 |
| 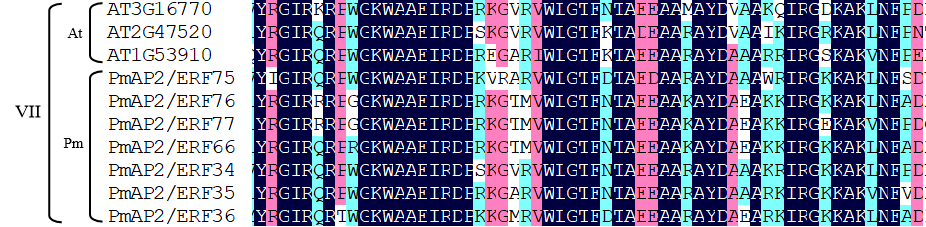 |
| 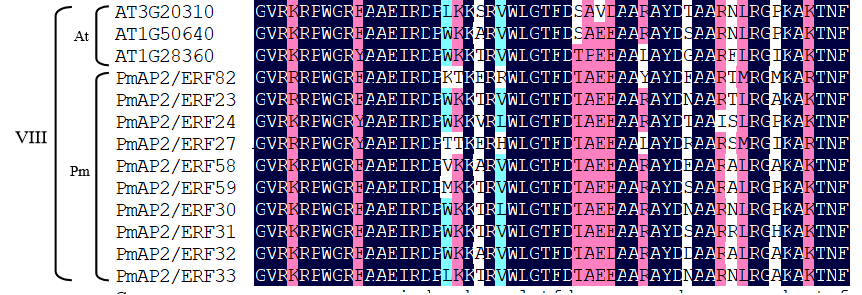 |
| 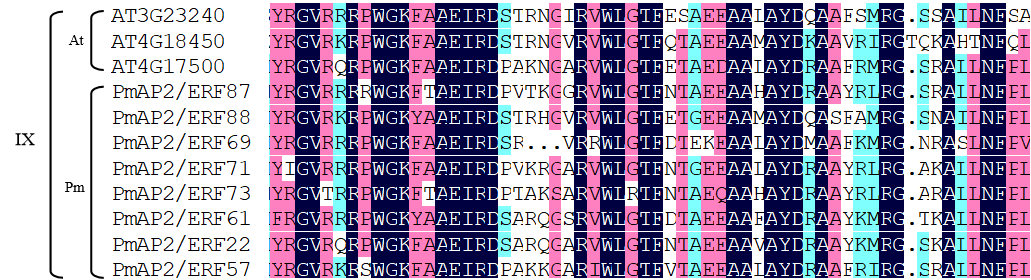 |
| 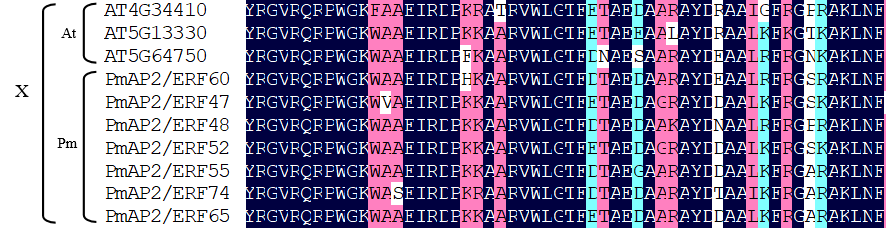 |
| 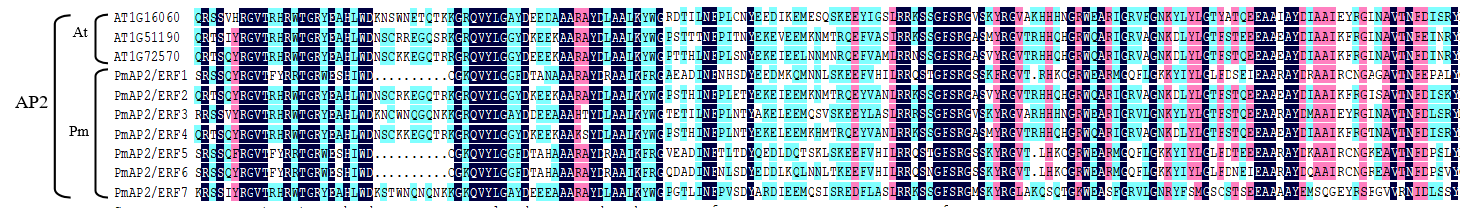 |
| 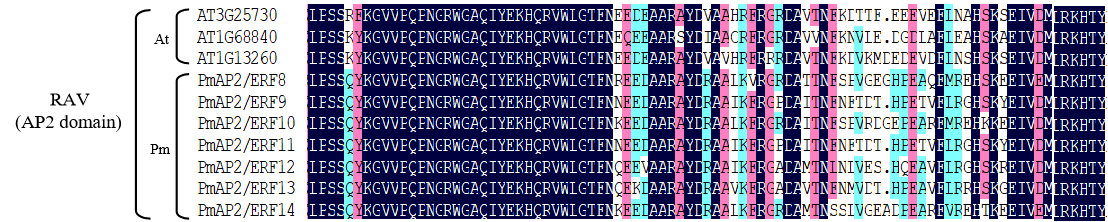 |
| 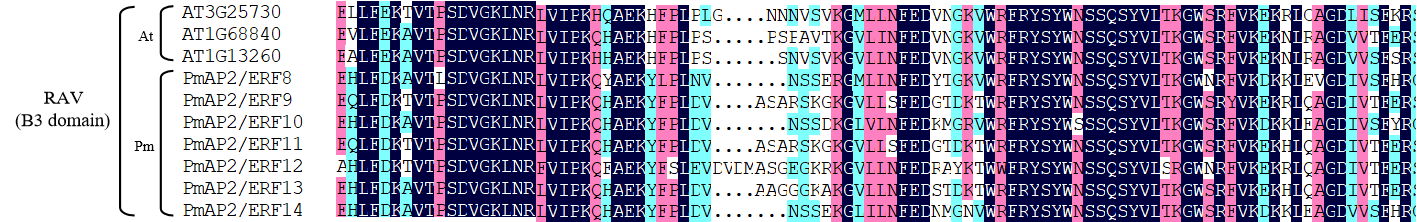 |
| **Figure S2.** Comparison of deduced amino acid sequences of the AP2 domain of the AP2/ERF subfamily proteins from *A. thaliana* (At) and *P. massoniana* (Pm)*.* |

| 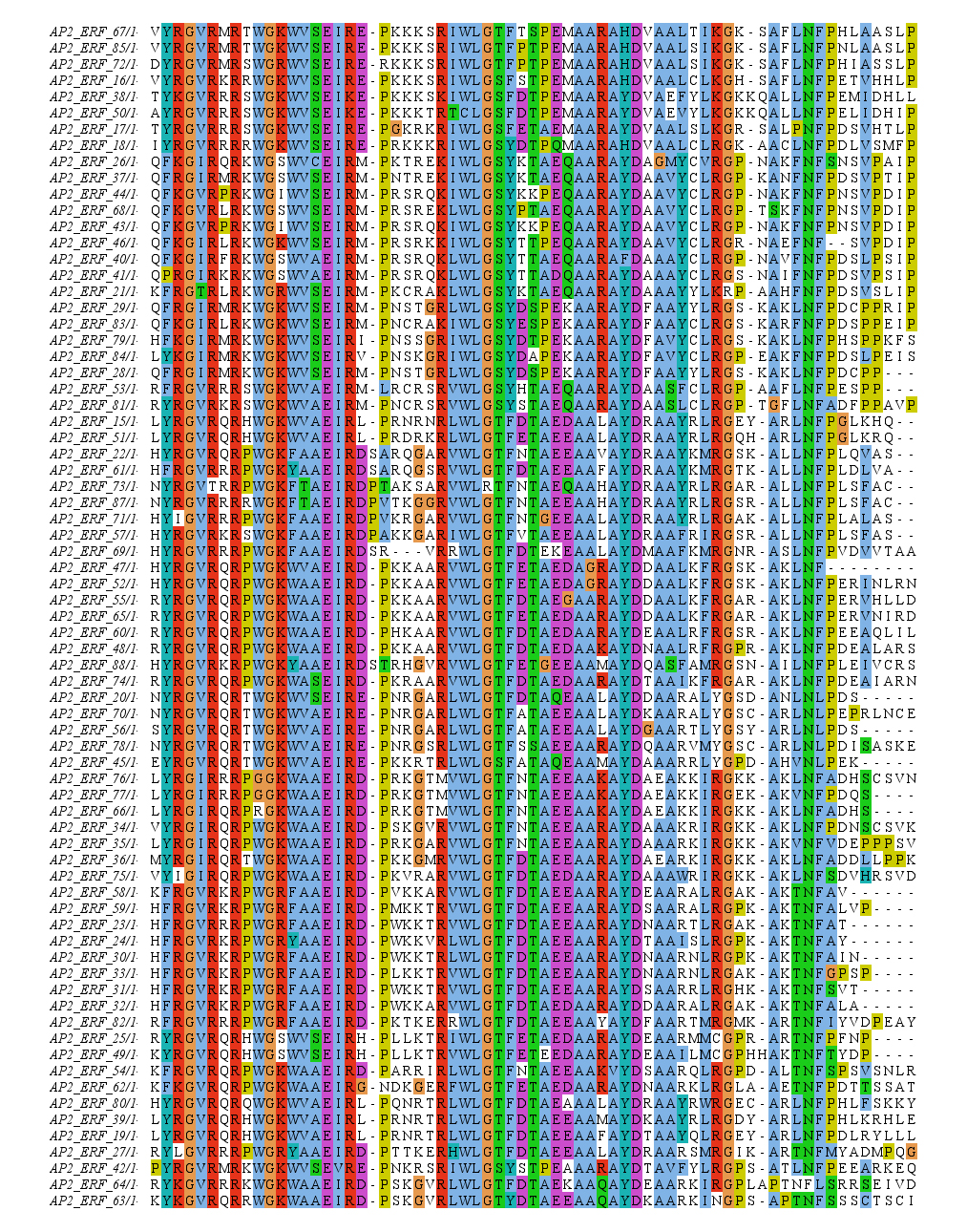 |
| --- |
| **Figure S3.** Sequence alignments of AP2 domains of all ERF family proteins in *P. massoniana*. |

| 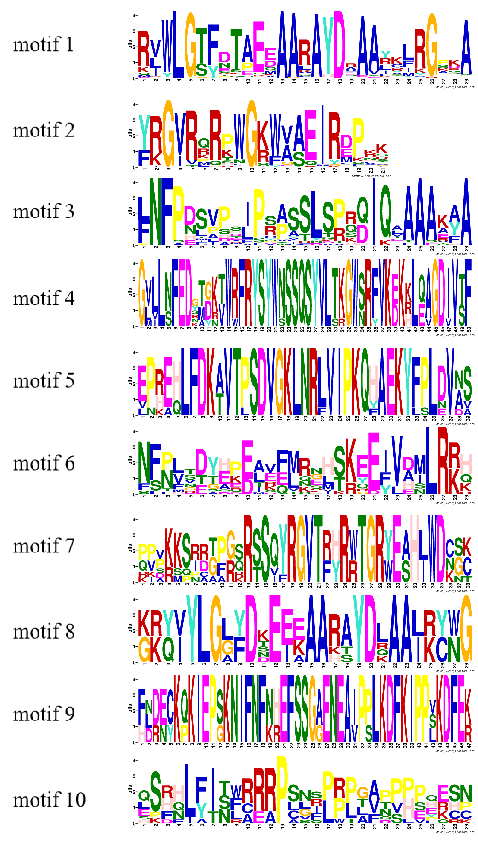 |
| --- |
| **Figure S4.** The ten conserved motifs identified in the AP2/ERF superfamily proteins. |
